# Health worker motivation in Africa: the role of non-financial incentives and human resource management tools

**DOI:** 10.1186/1478-4491-4-24

**Published:** 2006-08-29

**Authors:** Inke Mathauer, Ingo Imhoff

**Affiliations:** 1 German Technical Cooperation (GTZ), Eschborn, Germany

## Abstract

**Background:**

There is a serious human resource crisis in the health sector in developing countries, particularly in Africa. One of the challenges is the low motivation of health workers.

Experience and the evidence suggest that any comprehensive strategy to maximize health worker motivation in a developing country context has to involve a mix of financial and non-financial incentives. This study assesses the role of non-financial incentives for motivation in two cases, in Benin and Kenya.

**Methods:**

The study design entailed semi-structured qualitative interviews with doctors and nurses from public, private and NGO facilities in rural areas. The selection of health professionals was the result of a layered sampling process. In Benin 62 interviews with health professionals were carried out; in Kenya 37 were obtained. Results from individual interviews were backed up with information from focus group discussions. For further contextual information, interviews with civil servants in the Ministry of Health and at the district level were carried out. The interview material was coded and quantitative data was analysed with SPSS software.

**Results and discussion:**

The study shows that health workers overall are strongly guided by their professional conscience and similar aspects related to professional ethos. In fact, many health workers are demotivated and frustrated precisely because they are unable to satisfy their professional conscience and impeded in pursuing their vocation due to lack of means and supplies and due to inadequate or inappropriately applied human resources management (HRM) tools. The paper also indicates that even some HRM tools that are applied may adversely affect the motivation of health workers.

**Conclusion:**

The findings confirm the starting hypothesis that non-financial incentives and HRM tools play an important role with respect to increasing motivation of health professionals. Adequate HRM tools can uphold and strengthen the professional ethos of doctors and nurses. This entails acknowledging their professionalism and addressing professional goals such as recognition, career development and further qualification. It must be the aim of human resources management/quality management (HRM/QM) to develop the work environment so that health workers are enabled to meet their personal and the organizational goals.

## Background

The current human resources shortage in the health sector – mainly of sub-Saharan African countries – threatens the realization of plans for scaling up interventions to control the spread of diseases such as HIV/AIDS, malaria and tuberculosis. The *World development report 2004 *[1] states it clearly: Without improvements to the human resources situation, the health-related Millennium Development Goals cannot be achieved. The problems are multiple, the most serious being staff shortages, particularly in rural and remote areas. In many countries, the effects of insufficient capacity development in the health system are aggravated by migration and a mounting burden of disease. The *World health report 2006 *gathers ample evidence of the human resource challenges, but also provides ways forward to address the problems [2].

With respect to existing human resources, the low level of health worker motivation has often been identified as a central problem in health service delivery. For example, the results from a survey undertaken by the Gesellschaft für Technische Zusammenarbeit (German Technical Cooperation, GTZ) among representatives of ministries of health and GTZ staff from 29 countries showed that low motivation is seen as the second most important health workforce problem after staff shortages. From the perspective of health professionals, the challenges include lack of equipment, frequent shortages of supplies and a mounting workload – all these exacerbated in small and rural facilities. Furthermore, despite decentralization efforts, key functions of human resource management (recruitment, overall staff distribution, remuneration, promotion and transfers) remain highly centralized.

Despite interest in the issue of human resources for health, human resource management and the question of what can be done to strengthen health worker motivation in developing countries has so far not received as much attention as the subject merits.

There is a small but growing body of qualitative studies looking at motivation of health workers in developing countries that indicate the limitations of financial incentives on motivation and that reveal the importance of non-financial incentives [3-7]. A study in South Africa on the effects of a newly introduced, so-called "rural allowance" showed the limited impact on retention and motivation [8]. Similarly, analysing the role of wages in health worker migration, Vujicic et al. [9] conclude that what they call non-wage instruments may be more effective in reducing migration flows, as portrayed in a WHO report [10]. The study of Kingma [11], while undertaken in developed countries, also provides important insights on the limited effect of financial incentives on nurses and instead points at the relevance of non-financial incentives for nurses' job satisfaction and self-esteem.

In their study on health workers' motivation and performance in Benin, Alihonou et al. [12] suggest introducing non-financial incentives while also improving structural conditions. Stilwell [13] shows, by reference to Zimbabwe, that health workers based in remote areas, despite lack of financial incentives and hard working conditions, frequently exhibited a high level of motivation to perform well. She traces this motivation to good leadership and supportive management, among other factors. Her analysis suggests that certain non-financial incentives can have a beneficial effect on motivation, even under adverse conditions of insufficient pay and equipment, understaffing, etc. In a review of theories and empirical evidence of health workers motivation, Dolea and Adams [14] equally stress the importance of non-financial incentives.

Low motivation has a negative impact on the performance of individual health workers, facilities and the health system as a whole. Moreover, it adds to the push factors for migration of health workers, both from rural areas to the cities and out of the country [10,15]. It is therefore an important goal of human resources management in the health sector to strengthen the motivation of health workers, from heads of health facilities to auxiliary staff.

Financial incentives are important, and the problem of low salaries must be addressed, especially in situations where income is insufficient to meet even the most basic needs of health professionals and their families. But the evidence suggests that increased salaries are by no means sufficient to solve the problem of low motivation. More money does not automatically imply higher motivation. We therefore suggest that any comprehensive strategy to maximize health worker motivation in a developing country context has to involve a mix of financial and non-financial incentives.

To support the development of effective strategies for human resources management and the improvement of health worker motivation, the German Technical Cooperation (GTZ) has assessed motivation and motivational determinants in rural districts of Benin, Kenya, El Salvador and Nicaragua. The study aims at determining the role of non-financial incentives for motivation. This paper here presents the findings from the Benin and Kenya case studies.

The next section outlines the conceptual framework used, followed by a description of the methodology. The fourth section focuses on health workers' feelings, views and perceptions and opens the black box of the motivational process. The paper then analyses the current problems and potentials of selected HRM tools with respect to their motivational effect on health workers. The final section offers conclusions and recommendations for the way forward.

This paper will show that health workers overall are strongly guided by their professional conscience and similar aspects related to professional ethos that keep them going. Many health workers are demotivated and frustrated precisely because they are unable to satisfy their professional conscience and impeded in the pursuit of their vocation due to lack of means and supplies at work and due to inadequate or inappropriately applied human resources management (HRM) tools. The paper also indicates that the way HRM tools are applied is characterized by severe pitfalls affecting the motivation of health workers.

## Conceptual framework: motivation, non-/financial incentives and HRM/QM

**Figure 1:**
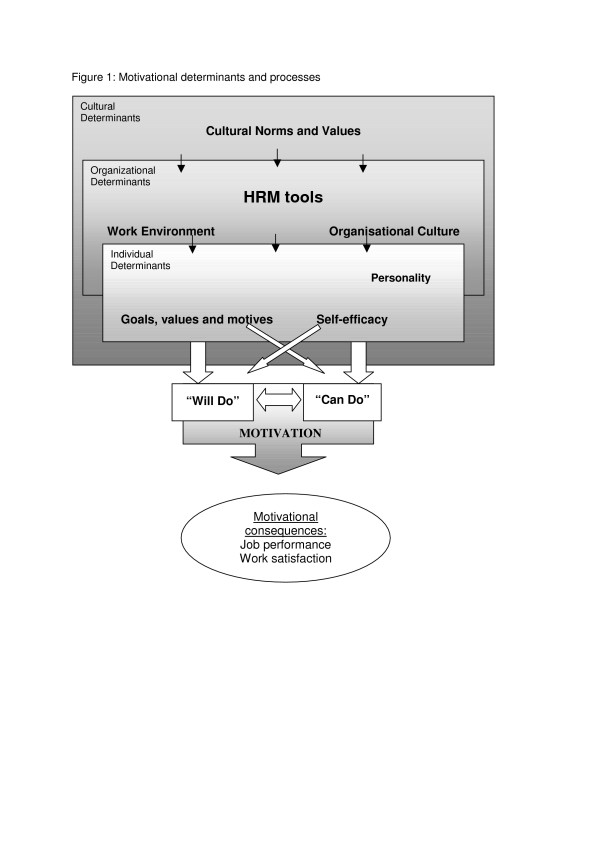
Motivational determinants and processes.

Motivation can be defined as "the willingness to exert and maintain an effort towards organizational goals" [16]. Motivation develops in each individual as a result of the interaction between individual, organizational and cultural determinants. Some of these factors are of more distal nature, such as cultural norms and values and individual personality, hence they lie outside the scope of human resources management. Using Franco et al.'s [4] terminology and concept, Figure 1 depicts how the various determinants influence each other.

Kanfer [17] identifies two aspects of the internal motivation process: The "will-do" aspect concerns the establishment of congruence between personal goals and the goals of the organization (goal setting). Questions that characterize this psychological process are: "What is the personal value of devoting more of my resources to the job?" or "What is the personal value of achieving higher job performance?"

The "can-do" aspect concerns motivational effectiveness, the extent of individual resources that are mobilized to accomplish adopted goals (goal achievement). The related question is: "How likely is it to achieve the desired level of job performance?".

The "will-do" and the "can-do" component can be distinguished for analytic purposes but the line between the two is blurred, as the findings of this study will show. Efforts to strengthen the motivation of doctors and nurses have to influence one or both of these components. Kanfer [17] and Franco et al. [16] do not explicitly mention a cross-impact between the "will-do" and "can-do" components, but the empirical findings of this study strongly suggest that there may be such an effect, which is indicated by the two crossing arrows in Figure 1. The result of this psychological process can be observed as motivational consequences with impact on the behaviour (job performance), affection (job satisfaction) and cognitive aspects (work attachment) of health workers. Kanfer's model offers a framework for the assessment of motivational determinants and the motivational process in greater detail, thereby going beyond the common and simpler distinction between motivators and satisfiers, as presented by Herzberg et al. [18].

In this study, we assess the impact of organizational determinants on the motivational process, specifically of HRM tools and non-financial incentives as part thereof. We define an incentive as an available means applied with the intention to influence the willingness of physicians and nurses to exert and maintain an effort towards attaining organizational goals. This is borrowed from Buchan et al., who define an incentive as "one particular form of payment that is intended to achieve some specific change in behaviour" (Buchan quoted in Adams and Hicks [19]).

The most common understanding of financial incentives is a transfer of monetary values or equivalents, such as wage increases, allowances, performance-related bonuses or housing. Adams/Hicks also include the basic salary, allowance schemes, health insurance premiums, housing or housing allowances in this category. Obviously, in practice it may be difficult to differentiate between the basic salary package and additional financial incentives. Non-financial incentives are by contrast those incentives that involve no direct transfers of monetary values or equivalents to an individual or group. This includes, for example, granting unpaid holidays, token awards or recreational facilities, as well as recognition and supervision [19].

Human resources management (HRM) is the management of people in an organization. HRM tools comprise the policies, practices and activities at the disposal of managers to obtain, develop, use, evaluate, maintain and retain the appropriate number, skill mix and motivation of employees to accomplish the organisation's objectives. There is a huge body of literature on HRM and tools, which cannot be captured here. But as the commentary on "what difference does ("good") HRM make?" by Buchan [20] shows, good HRM determines performance and motivation. In other words, tools and techniques applied as part of HRM also function as non-financial incentives to strengthen motivation in line with the above definition. The following list, derived from EFQM (European Foundation of Quality Management) criteria, contains some of the core HRM tools that may affect motivation. These aspects are similarly assessed in the *World health report 2006 *[2].

• supervision schemes

• recognition schemes

• performance management

• training and professional development

• leadership

• participation mechanisms

• intra-organizational communication processes.

The organizational culture influences all these aspects, but changes in these aspects may also modify the organizational culture. Furthermore, as outlined in Figure 1, work conditions constitute an important motivational determinant. Comprehensive HRM therefore also needs to look at and optimize the work conditions.

## Methods

The in-depth study design entailed (semi-) structured qualitative interviews with doctors and nurses from public, private and NGO facilities in rural areas. The questionnaire contained mainly open-ended questions. The interviews took about 60 to 90 minutes. Results from individual interviews were backed up with information from focus group discussions. For further contextual information, interviews with civil servants in the Ministry of Health and at the district level were held.

The qualitative interviews with health workers covered the following topics: prevailing practice; experience and views of health workers on core HRM/QM tools, such as training, supervision, performance assessments, participation, leadership, working environment, personal and career plans; and implicitly their effect on motivation. Some of the questions were not applicable for health workers who own a private clinic; these questions were therefore left out.

The last part of the interview focused explicitly on motivation and motivational determinants, yet the word "motivation" was avoided, except for two direct questions – "How do health workers define motivation?" and later "How do they assess their level of motivation?" – based on a given definition.

The study assesses motivational determinants and is concerned only indirectly with motivational consequences, to the extent that health workers report about these. An action research setting would have been required to assess motivational changes over time, triggered by the introduction or change of specific HRM/QM tools and (non-) financial incentives.

Before prospective respondents agreed to participate in the study, the interviewer informed them about the overall subject of the questions: their experiences and views of certain HRM tools and needs around their work environment. The actual research question on the role and relevance of non-financial incentives was not unveiled in order to avoid "socially desired behaviour" responses. The questionnaires were translated into French in a one-round de-centring process and then pretested. In each country, interviews were carried out by a consultant taking notes, rather than tape-recording. Qualitative data were coded centrally, thus avoiding inter-rater reliability concerns. The SPSS (Statistical Product and Service Solutions (formerly Statistical Package for the Social Sciences)) software package was used for the analysis of the quantitative and the coded qualitative data.

The sampling for the study was layered: The selection of districts was purposive in that it focused on GTZ-supported project areas, giving easy access to regional and district authorities. Facilities within a district were randomly selected on the basis of the district's facility list, except for the district hospital, which was always included. As the selection of facilities was based on the district's facility list, informal, non-registered private clinics were not part of the sampling process.

Random sampling on the basis of a staff list proved difficult. It was hard to arrange appointments prior to the interview, due to insufficient means of communication as well as staff absences. Ultimately, staff for the interviews were selected on the spot in cooperation with those in charge. Given the low numbers of doctors in rural facilities, every available doctor was interviewed.

A target of 50 interviews per country was set. Due to resource constraints, only 37 interviews could be held in Kenya. The Benin counterparts were interested in a larger sample and 62 interviews were realized. Except for the percentage of doctors (33% of the total sample should have been doctors), the sampling cornerstones were met: two thirds of interviews from the public sector, one sixth each from the private and NGO sector; at least one third of respondents to be female or male. No requirement was defined for the age of respondents. Table 1 provides an overview of the sample characteristics.

**Table 1:** Sample characteristics

		**Benin**		**Kenya**	
		**Number**	**Percentage**	**Number**	**Percentage**

**Number of respondents (N)**					
		62		37	
**Number of districts**					
		7		5	
**Professional background**					
	Medical doctors	7	11%	8	22%
	Nurses	55	89%	29	78%
**Age**					
	<25	3	5%	-	-
	25 – 34	30	48%	20	54%
	35 – 44	19	31%	10	27%
	45 – 54	9	15%	6	16%
	>55	1	2%	1	3%
**Sex**					
	Female	40	65%	14	38%
	Male	22	35%	23	62%
**Facility level**					
	Hospital	47	80%	22	59%
	Health centre	12	20%	15	41%
**Sector**					
	Public	41	66%	23	62%
	Private	10	16%	5	14%
	NGO/Mission	11	18%	9	24%

The study questions touched potentially delicate and personal issues relating to health workers' behaviour and attitude. Particular attention was therefore paid to triangulation. One means used was the knowledge of national and international GTZ staff involved in this study, who have detailed knowledge and long experience in the field that enables them to provide "external" assessments of certain problems and situations. Likewise, the focus group discussions reflected individual responses or drew out some delicate topics even more explicitly. Construct validity was enhanced by the consecutive use of similar questions and the reliance on several indicators to measure the same phenomena.

Ultimately, in order to find out what can be considered "true", it is also necessary to take into account what people can be expected to say on the basis of their interest and motives. Respondents may have stressed in particular the difficulties and challenges they are confronted with at their workplace. One may also expect individuals who feel very demotivated to be reluctant to admit so to the interviewer.

Certain questions by their type or content pose larger reliability and validity concerns than others, namely those asking respondents to rank themselves on a Likert scale. Such findings must be taken with some caution. They were included to provide a general impression.

Whether health workers really tell the "truth" cannot always be fully clarified. When this is the case, we clearly point it out in the text. On the whole, health workers appreciated the interest of "outsiders" in their personal concerns and opinions. There was no indication that they held back their views and judgements.

Given the sampling procedures as well as the small sample size, quantitative statements are not claimed to be representative for the entire health workforce of a country. Despite these limitations, the strength of this study lies in its exploratory and qualitative character. It sheds much light on what is considered important by health workers. By giving them a voice, we find out what really matters to them and which instruments have the potential to increase their motivation and job satisfaction and thus, ultimately, performance.

## Results and discussion

### Country information

In both Benin and Kenya, the public sector is the greatest health service provider and employer of health workers. Both countries suffer from shortages in staff resources, inadequate skills and very low salaries, particularly for nurses and other lower cadres. For example, depending on the job group, Kenyan nurses earn today between KES 5757 (about USD 72) and KES 12 450 (about USD 155) per month.

In order to retain doctors in the public service and improve the situation in under-supplied areas, the Kenyan Government has introduced additional "extraneous" and "non-practising" allowances for medical doctors, dentists and pharmacists. For doctors who enter the public service with a basic wage of KES 11 690 (about USD 145) per month, the allowances in this job group amount to KES 25 000 (about USD 311) per month. This means that wages of doctors have de facto tripled. According to a key informant in the Ministry of Health of Kenya, this measure has so far attracted 500 doctors to seek employment in the Kenyan public service. While wages have therefore improved for this job category, the working conditions in government health facilities remain largely unchanged.

In Benin, a nurse earns around 40 000 XOF (USD 75). Although a variety of allowances are paid for various kinds of tasks or functions (responsibility allowance, risk allowance, remoteness allowances), it remains difficult to survive on just this income. Most health professionals therefore engage in other income-generating activities. Especially for doctors, it is much more attractive and profitable to stay in urban areas, which is why it is difficult to attract them to rural areas.

### Health workers' motivation: strong professional ethos mixed with frustration

#### Health workers' understanding of motivation

As mentioned above, motivation can be defined as the willingness to exert and maintain an effort towards attaining organizational goals. Yet, when health workers are asked about their definition of motivation ("What does being motivated mean to you?"), a different understanding emerges.

As Table 2 shows, over 50% of health workers in Benin equate motivation with prospective "encouragement" or retrospective "re-compensation", which is understood as making them work better. Out of these, one fourth explicitly mention financial encouragement. Another 40% consider "being motivated" as having the means and material to work, to get recognition, or other HRM tools, such as awards, supervision and good leadership. Hence, the majority understands motivation as a "motivator", i.e. an incentive, and not as a state of mind. Only 5% refer to motivation as the "willingness" or the "pleasure" to do one's work, similar to the above definition, from the literature, of motivation as an intrinsic process and state of mind. Not surprisingly, the latter group are mainly doctors and health workers in the private sector.

**Table 2:** Health workers' understanding of motivation

**Understanding of motivation**	**Benin, N = 62, in % **	**Kenya, N = 37, in %**
Encouragement or reward	51	19
Means and materials	11	8
HRM tools (particularly recognition)	29	35
Willingness/pleasure	5	16
Other	4	12

In Kenya, one fifth understand motivation as encouragement. However, there is a larger share of health workers who refer to that intrinsic state of willingness and pleasure to do one's work. This difference in the connotation between Kenyan and Benin respondents may be due to the fact that so-called "motivation allowances" ("primes de motivation") have been introduced in Benin, that may have changed the meaning of motivation from a state of mind to that of an incentive.

This understanding of motivation matches health workers' perceptions of motivational consequences: For the majority of Benin respondents (70%), motivating someone serves to improve work performance, while only 30% perceive motivation as a means to increase job satisfaction and job attachment. Again, for Kenyans, there is a stronger focus on the satisfaction element (48%). Especially in Benin, this view of motivational consequences may represent a legacy from previous decades, when job satisfaction and pleasure were not part of the prevailing organizational cultures. As one key informant put it, individual health workers were merely seen as a part of the production chain.

The introduction of a quality management system, which is currently under way in Kenya and partly in Benin, may provide the opportunity to gradually change this mindset and to give the word motivation a new meaning. Alternatively, it may seem necessary to avoid the word motivation altogether and use a different terminology, such as for example "boosting one's work spirit or work morale".

**Figure 2:**
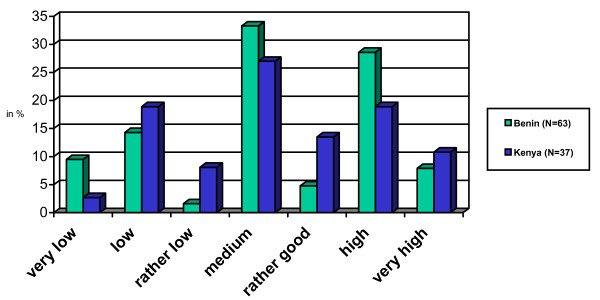
Self-assessment of motivation level.

In contrast to their own definition of motivation, health workers were asked to assess their level of motivation, by considering the following definition: "Willingness to do a good job, according to organizational objectives" (see Figure 2).

The health workers' replies suggest that they may not have responded in line with the given definition, but also referred to their own understanding of motivation. With the caveat that the validity of the answers may be somewhat limited, more so as the Likert scales for Benin and Kenya were not internally validated, the figure still provides an overall idea of how health workers see their own level of motivation: In both countries, more than 55% do not place themselves in the categories "rather good", "high" or "very high".

In Benin, health workers from the private sector/NGO facilities appear more motivated than those in public health facilities. Two thirds assess their level of motivation as "very high", "high" or "rather good" (14/21), compared to around a quarter from the public services (11/41). Nurses from the public sector appear to be the least motivated group and therefore a likely focus for efforts to increase motivation by means of human resources management and incentives. In Kenya, such clear tendencies cannot be derived, but then the sample size is also much smaller.

#### Motivational determinants: professional values and goals versus endangered self-efficacy

**Figure 3:**
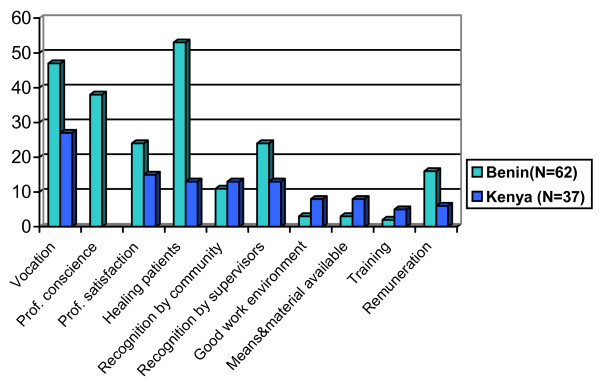
Aspects that encourage to do one's work well, in %, in Benin (N = 62), Kenya (N = 37).

To identify the motivational determinants that account for perceived level of health worker motivation, respondents were asked: "Which aspects currently encourage you to undertake efforts to do your work well?" (Figure 3). As Benin respondents gave longer answers, mentioning several aspects, percentage figures in Figure 3 are higher for Benin.

Health workers in Benin strongly referred to vocation and professional conscience, i.e. their personal professional values. Likewise, the wish to help patients and professional satisfaction were frequently mentioned. Pangu [21] carried out a study in Benin on the reasons why health workers stay in function despite decreasing motivation and identifies exactly those four aspects. Among Kenyan respondents, vocation was equally very dominant. Also, healing patients, professional satisfaction and recognition were considered important. These aspects nurture health workers' goals. Both dimensions – values and goals – indicate a strong professional ethos and commitment and strongly appear to translate into the "will-do" component of the motivation process.

Other factors, such as work environment and HRM tools that relate more to the "can-do" component are also mentioned, but particularly in Benin they do not feature as prominently as values and goals. About 6% of respondents in Benin and Kenya mention regular salaries and allowances. Finally, half of the health workers in the private sector in Benin, that is 10% overall, consider that earning revenue is an important encouragement to do one's work well.

**Figure 4:**
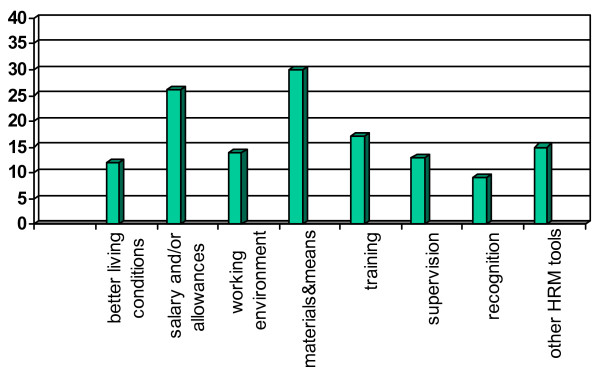
Benin: "How to boost your spirit and willingness to perform" (in %, multiple responses, N = 62).

When health workers were asked, with respect to the future, what would have to happen so as to boost their spirit and increase their willingness to perform (this was asked only of Benin respondents, see Figure 4), they emphasize to be able to perform one's work, namely having the materials and means available as well as further training and supervision. This corresponds with the findings of Alihonou et al. [12], as well as with a staff survey from Zimbabwe cited in USAID [22], which revealed that the number one reason provided by health workers for resigning their government job was the lack of equipment and supplies.

Also, a more conducive working environment and atmosphere, recognition and feedback, and other HRM tools such as better leadership, management and participation, are reported to contribute to increasing health workers' self-efficacy, i.e. their self-perception of being able to do a good job and fulfil their duties ("can-do" component of motivation). It also emerged from the interviews that health workers appreciate small benefits as relevant to their motivation. For example, in public health facilities, unpaid holidays or small amenities such as free tea for staff on night duty were perceived as motivating. Their absence, temporary or permanent, was considered demotivating.

The two questions on current versus future motivational determinants accordingly revealed different answers: Several aspects that featured low under the question on current motivational factors – precisely due to their (perceived) non-existence or poor state, such as organizational factors – then emerged in the second question as potential motivating factors.

Table 2 and Figures 3 and 4 also reveal that health workers self-evidently desire better salaries and living conditions, including better communication facilities. These aspects do not dominate, however. Instead, very prominent were health workers' request and need for available means and materials to carry out their work in a professional way. Subsequent questions, e.g. how health workers could improve their performance, persistently revealed the frustrations that health workers experience because of the lack of means and materials and the inadequate work environments as well as deficits in HRM.

It appears that these frustrations are so strong precisely because of the high professional commitment of health workers. In fact, there is a danger that perceived self-efficacy, constrained by inadequate HRM and work environment, becomes so weak that it may ultimately negatively affect health workers' commitment and professional ethos.

Health workers' commitment and professional ethos is, moreover, endangered by the existence of HIV/AIDS, which, as for example Aitken and Kemp [23] have shown for Southern Africa, has a severe impact on health workers' behaviour and above all attitude to work. In fact, throughout the interviews, health workers refer to HIV/AIDS issues. Asked about their feelings and reactions with respect to HIV/AIDS at the workplace, few health workers feel sufficiently protected. Most health workers fear becoming infected and report reacting in general more reserved towards patients. Some respondents said that every patient is seen as a potential source.

The next section assesses some core HRM tools and derives their effect on the "will-do" and "can-do" component of motivation. Respondents were asked about their experience with and perceptions of certain non-financial incentives and HRM tools. The findings reveal present shortcomings, but also reveal further potentials in the application of such tools, which is supported by the vast evidence and examples collected by the WHR 2006 [2].

### HRM tools: current pitfalls and further potentials

#### Supervision as control versus support supervision and recognition

Ideally, supervision is a formalized HRM instrument to correct shortcomings and to support good practice, on the basis of which recommendations are provided to help improve individual and facility performance. Supervision can contribute considerably to health workers' self-efficacy and relates therefore to the "can-do" component of Kanfer's model of motivation. To the extent that supervision is used to communicate a facility's goals and that it takes account of health workers' personal goals and needs, it also strengthens goal coherence and affects the "will-do" component of motivation.

The interviews revealed that 40% of respondents from Benin and more than 50% from Kenya perceive supervision as an exercise of control. As the following quotes reveal, existing schemes for supervision are sometimes perceived as unhelpful and distant, rather than personal and supportive:

"Supervision is not very useful. The supervisors remind nurses of the procedures they should apply. Right now, under given circumstances, you cannot implement them. They remind you of the rules and control you." (female nurse, 32 years, government facility, Kenya)

"While individual efforts go unnoticed, mistakes or shortcomings are noticed immediately." (female nurse, government facility, Kenya, in focus group discussion)

"The supervision went without difficulties, but the supervisors did not give me any feedback afterwards." (male nurse, 33 years, private facility, Benin)

Health workers criticize the low frequency and irregularity of supervision as well as the top-down approach used by supervisors. Supervision that involves discussions of health workers' conduct in the presence of patients is seen as particularly demotivating. Responses revealed that not knowing whether or when the next supervision takes place can also have a negative effect on health workers' commitment to improve their work.

In Kenya, almost half the respondents and one in ten of the respondents in Benin claimed that they do not receive any personal feedback from their superior. Judging from the answers provided, the feedback that health workers receive from their supervisors in rural facilities usually centres on specific shortcomings or technical aspects of service provision. It rarely appears to focus on the personal perspective of the health worker herself or himself. Feeling neglected by the superiors or the health administration has a strongly demotivating effect. There are indications that supportive supervision, recognition and personal feedback tend to be more common in religious and private health centres in Kenya than in public facilities.

Despite these shortcomings, health workers consider supervision useful and desirable to the extent that it helps improve personal performance, to avoid mistakes and to update knowledge. When personal needs and concerns are taken seriously, supervision provides the feeling of being cared for and of appreciation. This aspect appears particularly important for health workers posted to remote facilities with little contact with other professionals.

There is a large potential for improvement in the supervision process, according to the respondents' suggestions: Apart from a more frequent, regular and reliable supervision, many respondents ask for a different approach, in that supervision should be more supportive, instructive, needs-oriented, participatory and should provide a direct and timely feedback on their problems. There was also a call for more meetings to discuss problems and solutions. Direct observation of health worker activities is considered helpful to identify the bottlenecks in service delivery, provided that health workers are not criticized in front of the patients.

In conclusion, support supervision that exists appears to contribute significantly to health workers' self-efficacy. There are potentials to strengthen the "can-do" component of motivation through a more regular supervision routine and follow-up. Moreover, supportive supervision that takes account of the supervisee's personal and professional goals of recognition and learning and that communicates organizational goals clearly offers a potential for a positive motivational effect both in the "will-do" and "can-do" component that is so far not sufficiently used.

#### Lack of recognition versus institutionalized recognition and appreciation by superiors and communities

Through an encouraging and supportive attitude, superiors can strengthen their subordinates' self-efficacy and thus foster personal efforts for the achievement of organizational goals: the "can-do" component of motivation. Community recognition and appreciation can have the same effect.

While most felt that their leaders are accessible, critique focused on lack of encouragement and insufficient consideration of staff views, as health workers did not feel adequately supported and recognized by their superiors. In other words, the leaders seemed to fail to contribute as much as possible to strengthening health workers' self-efficacy. In general, health workers criticized their leaders' inadequate communication and bad treatment of staff. When leaders fail to be role models by not adhering to organizational goals, health workers may wonder why they should adopt them, thereby reducing the drive for the "will-do" component. In fact, a good number of respondents wished for supervisors to receive training on management and leadership issues. Clearly, health workers highly value recognition and appreciation from superiors and colleagues as well as patients. The role of good working relationships with superiors and colleagues similarly emerged as one important motivational determinant in a study in Georgia by Bennett et al. [7].

**Figure 5:**
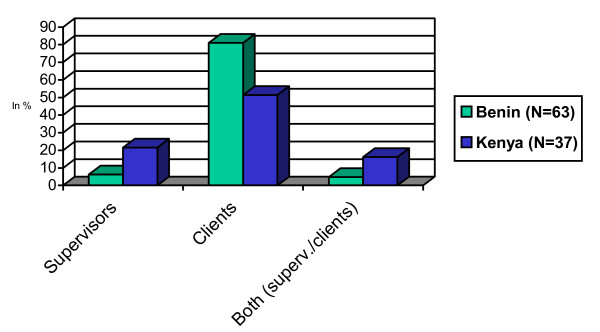
Preferred source of appreciation.

However, asked whose appreciation health workers find most relevant, 80% of Benin and 50% of Kenya respondents referred to the patients' appreciation (see Figure 5). There is no correlation by type of institutions, hence client appreciation is not of greater importance to health workers from the NGO and private sector than from the public sector. This is not to say that in practice, health workers are indifferent to their supervisors' appreciation. After all it is important for career promotion and adequate postings, but as Buchan [20] also notes, the "avowed first loyalty [of doctors and nurses] tends to be to their profession and their patients ..."

Appreciation from clients is seen as an indicator for successful professional conduct and the achievement of the health workers' goal to cure patients. The importance of patient appreciation stems primarily from health workers' search for professional satisfaction and their professional goals of helping patients, as revealed throughout the interviews, for example:

"Appreciation from the patients is most important, because it is sincere and heartfelt." (male medical officer, 28 years, government facility, Kenya)

The above finding strongly supports ideas and efforts for strengthening community participation in health service provision. Client satisfaction surveys, community dialogue and interaction with health unit management committees should be managed in such a way as to not only ensure client orientation and accountability towards the community but also to convey appreciation and strengthen staff motivation. Such improved mechanisms may also help to reduce health workers' perceptions of too much interference by the community, lack of patient compliance or lack of cooperation by patients and their relatives. These issues formed frequent complaints by health workers and appeared to affect the "can-do" component of their motivation.

#### Inadequate training versus needs- and problem-adapted training

Training and professional progress are important motivational determinants, as they nurture health workers' personal objectives and their value system. In fact, training as a tool of human resources management can serve several purposes. It can help health workers to cope better with the requirements of their job. It can also enable them to take on more demanding duties and positions and to achieve personal goals of professional advancement. Training can have strong motivating effects.

When asked about the effect of training courses they had taken over the past two years, nearly all health workers in Kenya and Benin mentioned that they felt more comfortable and confident with their work afterwards. Some 20% also mentioned increased interest and work commitment. These answers suggest a considerable effect of training courses on both the "can-do" and the "will-do" component of motivation. Overall, respondents were very interested to receive more continued medical education/continued professional development. This is similar to other study findings [7,24]. With respect to the contents required to fill gaps in their knowledge, they mentioned in particular training in management and administration as well as HIV/AIDS treatment.

The need to learn how to deal safely with HIV/AIDS patients through training in counselling and prevention was a recurrent theme among health workers. Basic training on HIV/AIDS prevention and care as well as having the means for care available appears to be a critical measure to help health workers maintain their morale and motivation, as indicated by such typical quotes as below:

"I have worked as a nurse before it [AIDS] came. Initially, I had a negative attitude to AIDS patients. There were many programmes. Now I am trained, so I have no more fear." (male nurse, 46 years, government facility, Kenya)

"Training changed me. It made me accept all patients and to share their feelings. I have no fear of patients anymore, I am more confident. I came back with new energy." (female nurse, 45 years, government facility, Benin)

Training may constitute a direct means to mobilize the motivational potentials inherent in the professional ethos of health workers. However, many respondents described the effect of training as short-lived or even frustrating:

"The training discouraged me because of the lack of equipment here. You cannot practise the information you were taught." (female nurse, 30 years, government facility, Kenya)

"The good feeling is gone in the government immediately. We don't have ultrasound. So the knowledge wears off." (male clinical officer, 30 years, government facility, Kenya)

Contents of courses and seminars that were out of touch with the reality of the rural health facilities of developing countries were considered the most important problem by health workers. After a training course, those confronted with treatment guidelines based on standards of equipment and supplies that do not exist at their workplace experience their daily work environment as an insurmountable obstacle to the provision of "adequate" health care. Improvisation to make up for shortcomings in equipment and supplies was considered inferior practice.

Overall, training can have a strong effect not only on the "can-do" component, but also on the "will-do" component of motivation. Yet, to have this effect, i.e. to exploit this motivational potential, training courses must be adapted to the local context: the actual working conditions in rural facilities.

#### Non-transparent allocation versus equal opportunities for training and professional progress

Further education and professional progress rank highest among the professional objectives of the nurses and doctors interviewed in Kenya and Benin. These objectives were mentioned by about two thirds of respondents. The answers reveal that the interest in further education is motivated by the chance to rise within the hierarchy of the health system, to reach a higher status and to increase earnings.

In Kenya, the prospect of sponsorship for further qualifications in the public health system appears to make public sector jobs more attractive for health workers working in private and mission facilities, although working conditions were acknowledged to be better in some of the private facilities.

Health workers in the public sector, by contrast, often feel demotivated by the limited realistic prospects of professional progress and personal advancement and the rather slow and cumbersome promotion process. Where access to training and further qualifications is limited or granted in line with reasons that are not equitably available or merit-based, this can likewise have detrimental consequences for the motivation effect of training as a tool of human resources management. The following quotes reveal that this results in dissatisfaction:

"It is necessary to make everybody participate in training opportunities, not always to privilege the same." (female nurse, 31 years, government facility, Benin)

"There is a problem with staff motivation. You work for four or five years in the same place. You find the same person goes to the same seminar in two years; why not rotate and train someone else the next year?" (nurse, government facility, Kenya, in focus group discussion)

Opportunities for training must be allocated in a transparent and fair way, since a sense of unequal treatment demotivates and leads to frustration. As such, it affects both the "will-do" and "can-do" component of motivation.

The use of training and further qualification as a human resources management tool is limited by financial resource constraints, of course. However, as individual examples show, there may be options for gradual improvement and needs-adapted training even in resource-poor rural settings. In Kenya, whereas the management of one facility ruled out Continued Medical Education for lack of funds to invite teachers, in another facility, the medical superintendent provided short information leaflets to update staff on basic guidelines and procedures. Given that 10% of health donor funds are spent on training activities, there is a great need – but also opportunity – in policies that address these deficits.

#### Passive staff involvement versus active staff participation

Staff participation and staff involvement is an important HRM tool. It permits making the best use of health workers' knowledge, hands-on experience and ideas for improvements. In addition, staff participation involves recognition and appreciation of health workers and their competences. It appeals to their need to be taken seriously. This has important implications for motivation, both for the "will-do" component (e.g., self-realization, professional satisfaction) and the "can-do" component (e.g., self-confidence, improvements around the workplace by taking account of health workers' knowledge and ideas).

Most respondents in Kenya and Benin felt they could contribute ideas and efforts, particularly with respect to daily activities. While Kenyan respondents seem to see this happen primarily through staff meetings, Benin respondents rather refer to their efforts on the job.

By contrast, 57% of Kenyan respondents felt they couldn't participate in decision-making at their facility, a perception shared by nearly 80% of Benin respondents. There was no difference between the different provider types. Still, nearly everybody considers participation as important and, with a few exceptions, the vast majority would like to be involved much more actively. The respondents indicate that the relevance of participation consists in sharing one's views and knowledge of a situation or of a problem-oriented solution, to be involved in decisions that concern oneself or to react where necessary in order to avoid frustrations.

Interestingly, under current conditions hardly any feeling of professional satisfaction or self-realisation among respondents appeared to result from participation. Staff involvement appeared to be a passive undertaking. It serves to collect ideas and proposals. It allows staff to get informed rather than to take part in actual decision-making.

Given that health workers are socialized in a rather top-down, hierarchical organizational culture within the public sector, which ultimately reflects cultural values and characteristics of the wider society, the idea of participation as a means of self-realization and particularly of recognition appears now to be hardly present. This is not to say that there is not potential for participation to serve as a tool for motivation. As one respondent said explicitly:

"Participation is very important because a decision taken concerning yourself has an important effect on your motivation." (female nurse, 30 years, government facility, Benin)

The findings suggest that increased participation could have an effect on both the "can-do" component (ideas of staff are taken into account to improve the work process) and the "will-do" component (influence on decisions to the benefit of health workers – satisfaction with respect to personal goals, appreciation of health workers' skills and competences – satisfaction with respect to professional goals). Hence, there is a great need to institutionalize participation, e.g. by holding regular meetings, in which health workers not only share ideas and suggestions for improvements, but in which health workers ultimately participate in decision-making on issues that concern their work and immediate work environment.

#### Infrequent performance appraisals versus culturally adapted performance management

Performance management tools serve to improve performance of health workers. Performance management assists in setting, communicating and internalizing organizational goals, thereby nurturing the "will-do" component. At the same time, this provides feedback to health workers as regards their capabilities and skills to achieve these goals, hereby addressing the "can-do" component. One specific tool is performance assessment, which this section looks at specifically. Other tools include the communication of the organizational mission, vision and objectives, self-assessments, quality improvement teams, performance related payments, benchmarking and awards. Performance assessment constitutes a review of a health worker's skills and achievements.

In Kenya, government health workers must pass an annual performance review. The interviews revealed that not everybody appears to know what this is about and which indicators are applied. Attitudes regarding the relevance of the annual performance assessments seem to be mixed. Some health workers – especially those with a very good report – say it is motivating. Also, several think the performance assessment leads to a change in practice and behaviour. Yet, some few say that it has no effect on one's practice and behaviour. Moreover, less than 30% (27%) state that the performance assessment can potentially have an impact on one's promotion. A good assessment is required for a promotion, and even though bad assessments appear to be rare, promotions are not forthcoming, as they are contingent upon vacancies and the availability of financial resources. Also, as one respondent says,

"It is a slow and painful process; you have to follow up on the promotion you are meant to receive in Nairobi." (female nurse, government facility, Kenya, in focus group discussion)

Furthermore, individual award schemes at the district level ("best unit" of district health facilities), although carried out on the basis of a detailed performance assessment, were considered unfair and thus demotivating by the staff, probably because the method and criteria of the selection process had not been sufficiently communicated.

In contrast to Kenya, there are no such performance assessments in Benin. Instead, Benin respondents from the public sector referred to the biannual supervision process that serves to monitor key indicators. After the exercise, all facility managers in a district get together to receive feedback, advice and recommendations from the district health authorities, as well as congratulations in some cases. Health facilities are ranked, which allows health workers to find out about their relative facility performance. The majority of respondents report that that they know the procedures as well as the indicators by which facilities are monitored. However, according to some respondents, the responsible facility managers do not always share the results with the rest of the team, which is considered frustrating or annoying. In practice, the facility-based supervision process seems to be translated to the level of individual health workers and can hence be understood as a form of performance assessment.

Several health workers mentioned that good performance may be rewarded through participation in training or by means of a scholarship. However, on the whole, respondents appear to see no direct impact of good performance on career development. One positive effect are the so-called "primes de performance" (performance allowances), which are paid for good indicator results. The public ranking and public congratulations appear to have a strong effect on health workers. They create competition and provide motivation to perform, as indicated by the following two quotes:

"For the public results, you fight for being the best." (female nurse, around 30 years, government, Benin)

"These evaluations are a source of motivation for us; if you get a good grade, [...] we make even greater efforts to keep our rank or to go even further." (female nurse with management function, around 40 years, government facility, Benin)

On the other hand, it is also mentioned that the public ranking exercise may be very frustrating for those at the lower end of the scale.

By providing feedback on health workers' work, performance assessments have some effect on the "can-do" component overall, since they help improve knowledge and skills. Yet this effect is currently small, as is the effect on the "will-do" component. Although the responses suggest that health workers are performance-oriented, the potential of performance management as a motivating instrument has not been not fully realized yet. Given the fertile ground for performance orientation, there appears to be scope for the gradual introduction of a performance culture, the search for excellence. The responses also suggest that health workers are receptive to performance assessments. Any initiative to promote a performance culture must take into account cultural values, norms and characteristics, as shown by the following section.

#### Disincentives for individual success versus team-based performance promotion

The findings reveal certain cultural factors that may impinge upon motivation and performance. Respondents were asked whether there are barriers that impede personal efforts and act as barriers to better performance. They were prompted specifically on envy among colleagues. The following quotes give flavour of the responses given in relation to envy:

"Making efforts on your own creates envy and you will face obstacles." (female nurse, 33 years, government facility, Benin)

"Individual efforts are not sufficient; you need to share your élan with others, otherwise you are badly looked at, and you will have the problems of your life." (young male doctor, government facility, Benin)

"If someone tries to take on an extra responsibility, others say 'they are making life more difficult for us.'" (male medical superintendent, 27 years, government facility, Kenya)

"If one colleague tries to work hard, others gang up against him." (male clinical officer, 33 years, government facility, Kenya)

In Kenya, nearly half the respondents stated that individual efforts are appreciated, while 30% confirm that envy indeed impedes individual efforts. In contrast, only a sixth of Benin respondents, among them all the private-sector doctors, feel it is possible and worthwhile to engage in individual efforts. Yet, nearly two thirds clearly expressed that individual efforts are futile and that team efforts are necessary to reach further. Half of the respondents mentioned envy as a barrier to individual efforts.

With respect to performance management, such feelings and views are problematic, because they may lead individuals to discount outstanding performance and the acceptance of responsibilities beyond theirs duties. The development of a performance culture, based on individual efforts, appears to be closely circumscribed by the social context. The findings indicate that it may be necessary to build performance management schemes upon group identities. This requires a focus on building teams and creating team spirit. Likewise, this suggests designing awards for groups rather than for individuals, more so as the latter creates conflicts within facility teams.

## Conclusion

### Supporting health workers and making them feel cared for: a call for quality management

The previous section has shown that health workers are strongly guided by their professional conscience and similar aspects related to professional ethos overall, relating to the "will-do" component of motivation. Many health workers appear to be demotivated and frustrated precisely because they are unable to satisfy their professional conscience and impeded in pursuing their vocation due to lack of means and supplies and due to inadequate or inappropriately applied HRM tools. These appeared to negatively affect the "can-do" component of motivation. Due to the extent of the problems at hand, they also affect the "will-do" component of motivation.

In conclusion, efforts to strengthen health worker motivation must protect, promote and build upon the professional ethos of medical doctors and nurses. This entails appreciating their professionalism and addressing health workers' professional goals such as recognition, career development and further qualification. It must be the aim of HRM and quality management to develop the work environment so that health workers are enabled to meet personal and organizational goals. This requires strengthening health workers' self-efficacy by offering training and supervision, but also by ensuring the availability of essential means, materials and supplies as well as equipment and the provision of adequate working conditions that enable them to carry out their work appropriately and effectively.

The findings confirm our starting point that non-financial incentives and HRM tools do play an important role when it comes to increasing motivation of health staff. The findings suggest that HRM tools have the dual task to promote health workers' professional ethos and commitment, and to strengthen their perception of self-efficacy.

The discussion of HRM tools above showed that there are serious deficits in their application, resulting in and reinforcing health workers' low motivation, primarily within the "can-do" component, but also affecting the "will-do" component. On the other hand, the analysis of health workers' responses and suggestions indicates that the HRM tools analysed above clearly constitute motivational determinants. In fact, the "toolbox" of HRM appears to have a great potential for improving health worker motivation. Also, only a small range of the available HRM tools have so far been applied in the countries that have been assessed.

The key message obtained from the interview data is the need to make health workers feel they are cared-for. Health workers require support in a variety of ways, ranging from technical aspects to personal and psychological needs. It is necessary to move to a culture of support supervision, to institutionalize recognition of health workers, to allocate training opportunities in an equal and transparent way, to fulfil health workers' drive for learning and acquiring new knowledge, to increase participation opportunities and to strengthen leadership. Ultimately, this requires changes in the organizational culture and individual attitudes, which can improve only by means of a gradual process in an enabling environment.

Therefore, greater awareness among superiors is needed in relation to their role as leaders. Leadership must become clear part of a superior's function and skills. It is important to note that good leadership is more than one single tool of human resources management; it is a fundamental and cross-cutting element interrelated with the style of participation, supervision and feedback, performance management and quality improvement activities.

The analysis also showed the applicability and usefulness of Kanfer's [17] and Franco et al's [4] differentiation into the "will-do" and "can-do" component, thereby shedding some light into the black box of the motivational process. Policy implications and recommendations therefore can become more specific and targeted. There is a different angle to it when we take high professional commitment as the starting point for a strategy of improving motivation. Furthermore, when contextualizing these findings into a quality management approach, there is additional impetus and additional implications as well as clearer guidance for action.

There is substantial overlap between HRM and quality management in terms of contents. Staff is considered and valued as a key resource in all major QM frameworks. QM is about leadership and HRM. It aims at improved management capacity of health workers and strengthens the capacity for participatory problem-solving.

A QM perspective and approach offers numerous benefits when it comes to optimizing human resources management. The QM framework permits developing, applying and evaluating HRM tools comprehensively, coherently and systematically. It offers a comprehensive set of tools that can be adjusted to fit specific circumstances and requirements – whether of a district, a hospital or a health centre. Furthermore, QM introduces the perspective of a continuous improvement process that looks at both outcomes and process. The process orientation ideally provides for continuous efforts to improve HRM under given circumstances.

Moreover, QM aims not only at changing and improving HRM tools and working conditions, it may also affect (or address) organizational values and hence organizational culture. QM is concerned with both organizational as well as individual determinants of motivation, while recognizing the importance of cultural values. The most widely used QM methods include self-evaluation and quality improvement projects, in which a team sets realistic objectives for process (and outcome) improvement and monitors the realization of the plan. Equally important are staff and client satisfaction surveys, as well as training.

While we do not aim to promote a specific quality management model, the following process requirements are considered essential for a contribution of QM to HRM:

• search for excellence: contribution of the whole staff body to quality improvement efforts;

• orientation on quality as an outcome as much as on quality of the process;

• strong emphasis on self-evaluation of individuals and organizations;

• more autonomy and responsibility for health workers;

• focus on participation and self-realization, empowerment and to a certain extent emancipation of health workers.

The findings indicate that the values on which QM is based are equally shared and referred to by health workers in the two countries under investigation. While certain HRM tools have become increasingly popular, also in African countries, the potential of QM seems far from exhausted.

### Recommendations and the way forward

Each country has a unique human resource situation, reflecting its stage in the health sector reform process. Accordingly, there is no overall blueprint on how to best improve health worker motivation. Each region, and even each facility, may require a specific mix of financial and non-financial incentives as part of a larger HRM or QM framework.

Findings from a survey on HRM experiences among health projects and programmes supported by GTZ in 29 countries (18 in Africa) suggest putting together a mix of incentives and HRM tools from the list in Table 3, aiming at both individuals' aspirations and organizational changes. These entry points can be realized incrementally even in resource-poor settings and make a difference when it comes to service delivery. This goes well hand-in-hand with the bundle of measures suggested by the *World health report 2006 *to enhance health worker performance [2].

**Table 3:** Incentives and HRM tools

**Introduction of and/or promotion of:**
• group-based performance awards and pay
• effort-related awards and pay
• consistent application of clearly defined sanctions for wrongful behaviour
• exposure to new knowledge (training, conferences)
• team building
• low-cost benefits that express personal appreciation (extra free time, tea during night duty)
• development of career development plans
• transparent and reliable promotion schemes
• continuing professional development, training
• supportive supervision and feedback
• performance management tools
• staff satisfaction surveys
• increased staff participation in decision-making processes within the health structure
• horizontal and vertical communication among staff
• quality improvement teams and building a quality culture
• participatory problem assessments and problem-solving processes
• benchmarking and competition among facilities.

Specifically with respect to performance management, there is a need to inform health workers about the process and indicators and to provide thorough feedback on the results. Also, consistently good performance should be the basis for promotional schemes by including elements of merit rather than length of service. This also necessitates streamlining the promotion process and making it more transparent. In Benin, the competition among facilities seems promising. However, within this ranking process, it is important to consider not only absolute scores, but also to take into account efforts and improvements over time. This is particularly relevant, as some facilities may suffer from much more serious drawbacks than others. Recognition and awards, which do not always have to be financial, appear to be highly appreciated. They should be applied in a systematic and transparent way.

Experience from the health projects supported by GTZ, which have relied on applying a mix of financial and non-financial incentives, shows that positive impacts can be achieved. For example, in Zambia, the introduction of refresher training for medical staff seems to have led to a higher retention rate. In Ethiopia a mix of continued medical education, the provision of housing, the establishment of a clear career structure and a defined number of services in hospitals has led to improved staff satisfaction and retention. An awards scheme, closer supervision and team-building efforts have improved both some service output indicators and the motivation to stay in rural districts of Ghana. As a result, higher antenatal care and EPI coverage as well as fewer applications for transfers have been reported.

These experiences call for a more systematic assessment. In fact, most countries, whether in the public or the private sector, in donor-supported programmes or NGO-run projects, apply a range of non-financial incentives. However, often this is not done systematically or specifically in relation to motivation and performance increases. As such, the impacts and effects of non-financial incentives are rarely monitored or evaluated. Hence there is great need to develop simple and practical methods to monitor the impact of incentives on staff motivation.

There are many challenges concerning the effective and sustainable implementation of incentive schemes and HRM tools, let alone comprehensive QM frameworks. With respect to allowances and financial incentives, the effect on staff motivation is often temporary in nature because such benefits become part of the general benefit package. Likewise, although training is seen as a good motivating instrument, high staff turnover may limit the sustainability of staff training.

Furthermore, there is a particular challenge with respect to introducing performance-related payment: It is difficult to define objective and commonly accepted performance criteria and even more difficult to implement and apply those performance criteria, given political interference and lack of good governance and transparency and the existence of corruption as well as nepotism.

In addition, it is very difficult to change old habits and long-term institutionalized norms and rules, such as promotion schemes that are based on age rather than merit. Another problem is caused by cultural norms that may negatively sanction an individual who is willing to show initiative beyond his or her duty, thus outperforming colleagues.

Hence, ultimately, HRM and QM measures must be imbedded in a good governance agenda in order to be effective and successful in the long run. Fostering transparency and accountability is necessary, since many of the factors that have a detrimental effect on health worker motivation are rooted in systemic or structural deficits. The role of donors and development partners is to strengthen institutional capacities at headquarters levels and at the district level to make a meaningful decentralization process, also in the field of human resources management, possible.

Likewise, we must be aware that the introduction and institutionalization of non-financial incentives and the various HRM tools or their improvement has its costs. It requires training, supervision from higher levels and follow-up. Change in organizational culture takes time. It is therefore important to develop realistic HRM plans and to provide for the financing to implement such measures.

Moreover, united action is needed from the donor community. The use and motivational effect of non-financial and financial incentive schemes in public health systems and private practice may remain limited as long as other development partners provide high top-up allowances as a financial incentive. Thus, donors should seek agreement on a code of conduct that establishes guidelines for orienting the development partners' activities – ranging from remuneration for health staff to sitting allowances, i.e. allowances paid for attending a training or a workshop.

Finally, we must be realistic: improved human resources management cannot compensate for many other factors, at both the macro and micro level, that seriously impinge upon work performance and staff retention, such as staff and supply shortages, heavy workload and difficult working conditions, migration pull factors originating from developed countries, as well as the threatening HIV/AIDS pandemic. Non-financial incentives and HRM/QM tools are not a magic bullet that solves the pressing HRH problem and compensates for the lack of investment and the structural deficits that characterize health systems in many low-income countries – there is no such magic bullet. However, as the discussion above indicates, these tools can make a difference and may be effective even in a resource-constrained context.

## Competing interests

The author(s) declare that they have no competing interests.

## Authors' contributions

IM developed the conceptual framework and the methodology, analysed the data, interpreted the results and drafted most of the manuscript. II contributed to the conceptual approach and the questionnaires as well as the data analysis, collected data in Kenya, and contributed to the manuscript. All authors read and approved the final manuscript.
